# Fast & furious: Rejecting the hypothesis that secondary psychopathy improves reaction time-based concealed information detection

**DOI:** 10.1371/journal.pone.0311948

**Published:** 2024-10-15

**Authors:** Imbar Mizrahi, Nathalie klein Selle

**Affiliations:** 1 Department of Criminology, Bar-Ilan University, Ramat Gan, Israel; 2 The Leslie and Susan Gonda (Goldschmied) Multidisciplinary Brain Research Center, Bar-Ilan University, Ramat Gan, Israel; Sapienza University of Rome: Universita degli Studi di Roma La Sapienza, ITALY

## Abstract

Deception, a complex aspect of human behavior, is inherently difficult to detect directly. A valid alternative involves memory detection, particularly through methods such as the Reaction-Time based Concealed Information Test (RT-CIT). The RT-CIT assesses whether an individual possesses specific knowledge by presenting various probe (familiar) items amidst irrelevant (unfamiliar) items. The task-required "unfamiliar" response to probes may induce a response conflict. Resolving this conflict, by inhibiting the automatic "familiar" response, takes time and slows probe RTs–a phenomenon known as the RT-CIT effect. Notably, secondary psychopathy is characterized by disinhibition and impulsivity, traits which may hinder the ability to effectively manage experienced conflict. Therefore, we hypothesized that secondary psychopathy would be associated with an elevated RT-CIT effect. To investigate this hypothesized relation, we conducted a pre-registered study (*n* = 86, student sample), employing a novel CIT paradigm that incorporates no-go trials to assess response inhibition capacity. Psychopathic traits were measured using the Levenson Self-Report Psychopathy (LSRP) scale, while the Barratt Impulsiveness Scale (BIS-11) assessed impulsivity. The novel CIT paradigm revealed impressive detection efficiency. However, contrary to our expectations, we observed no significant correlation between the RT-CIT effect and secondary psychopathic traits (BF_01_ = 6.98). This cautiously suggests that while secondary psychopathic tendencies do not improve RT-CIT validity, they also do not compromise it. Although future investigations should explore more diverse contexts and populations, this tentative finding is reassuring and underscores the robustness of the CIT paradigm.

## Introduction

Lying is an intrinsic feature of human behavior [1]. We all lie and we have all been lied to [2–4]. When people are asked to discriminate between truth and lie based on their perceptions, they correctly notice lies in about 47% of cases and classify truths as nondeceptive in about 61% of cases–which is close to chance level [5, 6]. Hence, it’s not surprising that throughout history humans have sought for techniques and methods that can distinguish between truth and lie [2, 7, 8]. In ancient Israel, for instance, a woman accused of adultery was considered guilty if her belly swelled after drinking "bitter water" [7]. In ancient China, those accused of fraud had to hold dry rice in their mouths–if the rice stayed dry, they were deemed guilty [9].

These historical methods, though lacking scientific validation, hint at a connection between physiological changes and deception. Building upon this understanding, psychophysiological methods for lie detection, popularly known as "polygraphs", emerged in the early twentieth century [10]. Such lie detection tools generally rely on physiological reactivity [11–13]. Importantly, the difference between the various lie detection methods lies in the adopted paradigm and the way its questions are formulated [see 12]. The classical, and probably most influential method, is the Control Question Test [CQT; 14]. This test assumes that guilty examinees will show stronger physiological responses to relevant, e.g., crime-related, questions, whereas innocents will show stronger physiological responses to control questions [15].

However, in real police investigations, both guilty (liars) and innocent (truth tellers) suspects may quickly identify the relevant questions and become emotionally aroused by them [11]. As a result, both types of subjects (guilty and innocent) may show enhanced physiological responses to the relevant questions, making accurate classification difficult [11, 16–19]. Consequently, it is not surprising that the scientific community has criticized this method for being biased against the innocent in addition to lacking a theoretical basis [15–17, 20]. Indeed, many criminal investigations have been hindered by the unreliable results of the CQT. For instance, consider the infamous Green River Killer case, which began in 1982 with the discovery of five bodies in the Green River, Washington. In this case, Melvin Foster, a taxi driver, failed a CQT despite his innocence. It wasn’t until 2001 that DNA evidence implicated Gary Ridgway, who was ultimately convicted of 49 murders. Remarkably, Ridgway passed a CQT in 1984 [19, 21–24].

Lykken (1959) was one of the first to question the existence of specific deception reactions and, hence, he developed the Guilty Knowledge Test [GKT; 25]. Today, the GKT is called the Concealed Information Test [CIT; 25] and is considered a well-validated diagnostic test that aims to detect concealed knowledge [11]. In this test, examinees are faced with several multiple-choice questions, each followed by one probe (e.g., crime-related) item and several irrelevant alternatives, which are similar to the probe [26]. For instance, in the Green River Killer case, the body of the first victim, Wendy Lee Coffield, was pulled from the river with a pair of blue jeans knotted around her neck [21, 22, 24]. An appropriate CIT question could have been: "What article of clothing was tied around the victim’s neck?" (a) black sweater; (b) purple shirt; (c) blue jeans; (d) red scarf; (e) green jacket. Importantly, knowledgeable suspects recognize the significant probes, leading to differential physiological and behavioral responses. Unknowledgeable suspects, on the other hand, cannot distinguish between probe and irrelevant items and respond uniformly to all items [12, 13, 27, 28]. Interestingly, while CIT researchers traditionally relied on autonomic physiological measures like heart rate, skin conductance, and brain responses, recent studies have incorporated behavioral measures such as reaction time [29, 30].

The RT-based CIT is designed according to the 3-stimulus protocol and includes, in addition to probe and irrelevant items, a third item type known as the “target stimulus” [31, 32]. These targets ensure stimulus-processing as they require a unique response [33, 34]. Specifically, participants are typically asked to judge the stimuli on familiarity and are instructed to press buttons with the captions "familiar" (for targets) versus "unfamiliar” [for probe and irrelevant items 35, 36]. The task-required "unfamiliar" response to probe items is presumed to create a response conflict [37, 38]. Such response conflict may be resolved by inhibiting the automatic “familiar” response, which requires time [39–41]. Hence, *response conflict* has been theorized to underlie the longer RTs for probe versus irrelevant items–i.e., the RT-CIT effect [28, 42, 43].

Several studies provide direct support for the role of response conflict. Suchotzki *et al*. (2018), for instance, reasoned that since conflict arises when one denies familiarity with the known probe items, conflict should be stronger when one relies more heavily on familiarity. To explore this hypothesis, the authors manipulated familiarity-based responding by: (1) increasing the number of different targets (4 instead of 2 newly learned targets); and (2) using more familiar targets (2 personally relevant instead of 2 newly learned targets). Both manipulations increased the RT-CIT effect, supporting the response conflict account. Moreover, Suchotzki *et al*. (2015) instructed participants to admit knowledge of half the probes and deny knowledge of the remaining half. Their findings showed that overt deception, which generates response conflict, was essential for both the RT-CIT effect and the activation of the right inferior frontal gyrus, a brain region associated with inhibition [44, 45]. Interestingly, a recent study has provided support for the crucial role of conflict, however, also suggests that additional factors such as orientation to significant information contribute to the RT-CIT effect [46].

Beyond theoretical considerations, meta-analytic research [29] has demonstrated that the RT-CIT is a highly valid method for detecting concealed information. Nevertheless, it remains to be assessed how the RT-CIT is affected by different personality traits, such as the constellation of traits associated with psychopathy [47, 48]. This is especially relevant considering that psychopathic individuals constitute a significant proportion of the incarcerated population, with prevalence ranging from 20% to 30% [49, 50]. Notably, classical dual-factor models of psychopathy distinguish between primary and secondary variants [51–54]. Secondary psychopathy, which is characterized by disinhibition and impulsivity, holds particular relevance in the context of the RT-CIT [55–59]. Specifically, a diminished ability to inhibit responses and manage response conflict should lead to an elevated RT-CIT effect.

Only a few studies have examined the influence of psychopathy on the CIT and found a significant CIT effect for psychopaths, which did not differ from that of non-psychopaths. However, these studies relied on physiological responses rather than RT [60–62]. RT serves as a behavioral measure and is assumed to reflect a different cognitive mechanism. Specifically, while the autonomic CIT effects have been tied to either orienting or arousal inhibition [see 63–66], the RT-CIT effect has primarily been associated with response conflict [28, 46]. As outlined above, efficient conflict resolution requires adept inhibition capacities, which may be compromised by secondary psychopathic tendencies [55, 57, 59]. Therefore, the objective of the present study was to examine whether the RT-based CIT is sensitive to secondary psychopathic traits in a student sample. To get a fuller comprehension of this relationship, we used a novel CIT protocol which features no-go trials to assess disinhibition (see Method).

## Method

This study was approved by the Ethics Review Board of the Criminology department of Bar-Ilan University (BIU; January 26^th^, 2023; see Ethics Review Board approval on https://osf.io/s5mrn/) and was performed in accordance with the relevant guidelines and regulations. The methods of this study, including sample size determination and exclusion criteria, were pre-registered on: https://osf.io/hz58u.

### Participants

A total of 100 BIU students (79% female) were recruited through BIU’s online research portal (i.e., SONA). Participants’ average age was 23.88 years (*SD* = 2.3, range = 20–37). All participants signed an informed consent form. At the end of the experiment, each participant received one credit point. All data of fourteen participants were excluded: thirteen participants were excluded because they made more than 50% errors to either target, probe or irrelevant items, and one participant was excluded because s/he did not complete the entire CIT (< 336 trials). Accordingly, the final sample included 86 participants (81.4% female, average age = 23.83, *SD* = 2.3, range = 20–37).

As indicated in the pre-registration, we stopped data collection when we reached *N* = 100, since the Bayes Factor (BF) provided substantial evidence for the null hypothesis (i.e., BF_01_ > 5; there is no linear association between the RT-CIT effect and secondary psychopathic traits).

### Materials

The present study included (1) the Levenson’s Self-Report Psychopathy (LSRP) scale, which provided the psychopathy scores; (2) a Go/No-go RT-CIT, which provided the RT-CIT effect as well as a behavioral measure of response inhibition (i.e., the no-go error rate; as explained below); and (3) the Barratt Impulsiveness Scale (BIS-11), which provided the impulsivity scores.

#### LSRP

Psychopathic traits within our student sample were assessed using the LSRP [67]. The LSRP contains a total of 26 items, rated on a four-point Likert scale from “disagree strongly” to “agree strongly", resulting in a total score range from 26 to 104. Developed specifically for non-forensic populations, the LSRP distinguishes between primary and secondary psychopathy, aligning with the original Psychopathy Checklist–Revised (PCL-R) factors [67–71]. The primary psychopathy subscale (16-items; range: 16–64) evaluates interpersonal and affective features of psychopathy, while the secondary psychopathy subscale (10-items; range: 10–40) assesses impulsivity and antisocial lifestyle [60, 67, 72].

The overall scale’s reliability typically falls within the range of 0.59 to 0.87; for the primary subscale Cronbach’s alpha ranges from 0.74 to 0.86, and for the secondary subscale, it ranges from 0.61 to 0.71 [67, 72–75]. In the current study, Cronbach’s alpha values were 0.79 for the overall LSRP, 0.8 for the primary subscale, and 0.63 for the secondary subscale. This study used a Hebrew translated version of the LSRP [76].

#### Go/No-go RT-CIT

The Go/No-go task is widely used in psychology as a measure of inhibition and impulsivity [77]. Therefore, the present experiment integrated this task within the RT-CIT–i.e., this study relied on a Go/No-go RT-CIT with both go and no-go trials. The regular CIT items–probes, irrelevants and targets–played the role of ’go’ items, to which participants had to respond by pressing a button. Specifically, a “unfamiliar” button for probes and irrelevants, but a “familiar” button for targets (as is common in the RT-CIT). When seeing the no-go items, participants were asked not to respond. Importantly, these no-go items were used to measure participants’ capacity for response inhibition, which is assumed to be compromised in secondary psychopathy [78, 79].

#### BIS-11

In addition to measuring response inhibition capacity with the novel no-go trials, we assessed impulsivity using the Barratt Impulsiveness Scale [BIS-11; 80]. The BIS-11 is a self-report questionnaire which contains a total of 30 items that are rated on a four-point Likert scale ranging from “rarely/never” to “almost always" [81]. Cronbach’s alpha for the BIS-11 typically falls within the range of 0.69 to 0.83 [80, 82, 83]. In the current study, Cronbach’s alpha was 0.84. This study used a Hebrew translated version of the BIS-11 [84].

### Procedure

The experiment was built in PsychoPy [85] and performed online in ’Pavlovia’ (see script on https://osf.io/s5mrn/). Participants received a link to the experiment through SONA (i.e., BIU’s online research portal). Importantly, once participants finished the experiment, SONA prevented them from performing the experiment again. The experiment contained three main stages: (1) the LSRP questionnaire, (2) the RT-CIT, and (3) the BIS-11 and subjective ratings. Before starting the experiment, participants read and approved an informed consent form (by pressing a button).

#### Stage 1

The LSRP questionnaire was completed after signing the informed consent form. All items (a total of 26) were presented one by one, and participants were asked to rate their agreement for each item, on a scale from 1 (“disagree strongly”) to 4 (“agree strongly").

#### Stage 2

Before starting the actual CIT, participants were presented with two item-lists, one of last names, and one of first names (female names for women and male names for men). Each list contained 16 items (i.e., names). Participants were asked to mark a maximum of 12 names, from each list, that have a special meaning for them. The irrelevant items (for the CIT) were chosen randomly from the words that were not marked.

Then, participants were explained about the upcoming CIT and motivated to conceal their autobiographical items (i.e., the probe items). To increase motivation, participants read a short paragraph which states that the upcoming task is difficult, and that only highly intelligent people with a strong willpower can successfully conceal. In addition, to become familiar with the no-go items, Tiger and Zebra, participants read a short paragraph about these items (i.e., Two animals with spectacularly beautiful stripes patterns are the Tiger (part of the Felidae family) with black-orange stripes, and of course, the Zebra (part of the Equidae family) with black-and-white stripes). Similarly, to become familiar with the target items, Caesarea and Milan, participants read a short paragraph about these cities (i.e., Who has not heard about the city of Milan, which is located in northern Italy and known for its great wealth? And of course, there is no one who does not know the city of Caesarea that was established 2000 years ago by the Roman Empire!). Thus, the CIT items were divided into three semantic categories, names for probes and irrelevants, cities for targets, and animals for no-go items. In total, there were 14 distinct items: 2 probes (participants first name and participants last name), 8 irrelevants (4 other first names and 4 other last names), 2 targets (Caesarea and Milan), and 2 no-go items (Tiger and Zebra).

**Fig 1:**
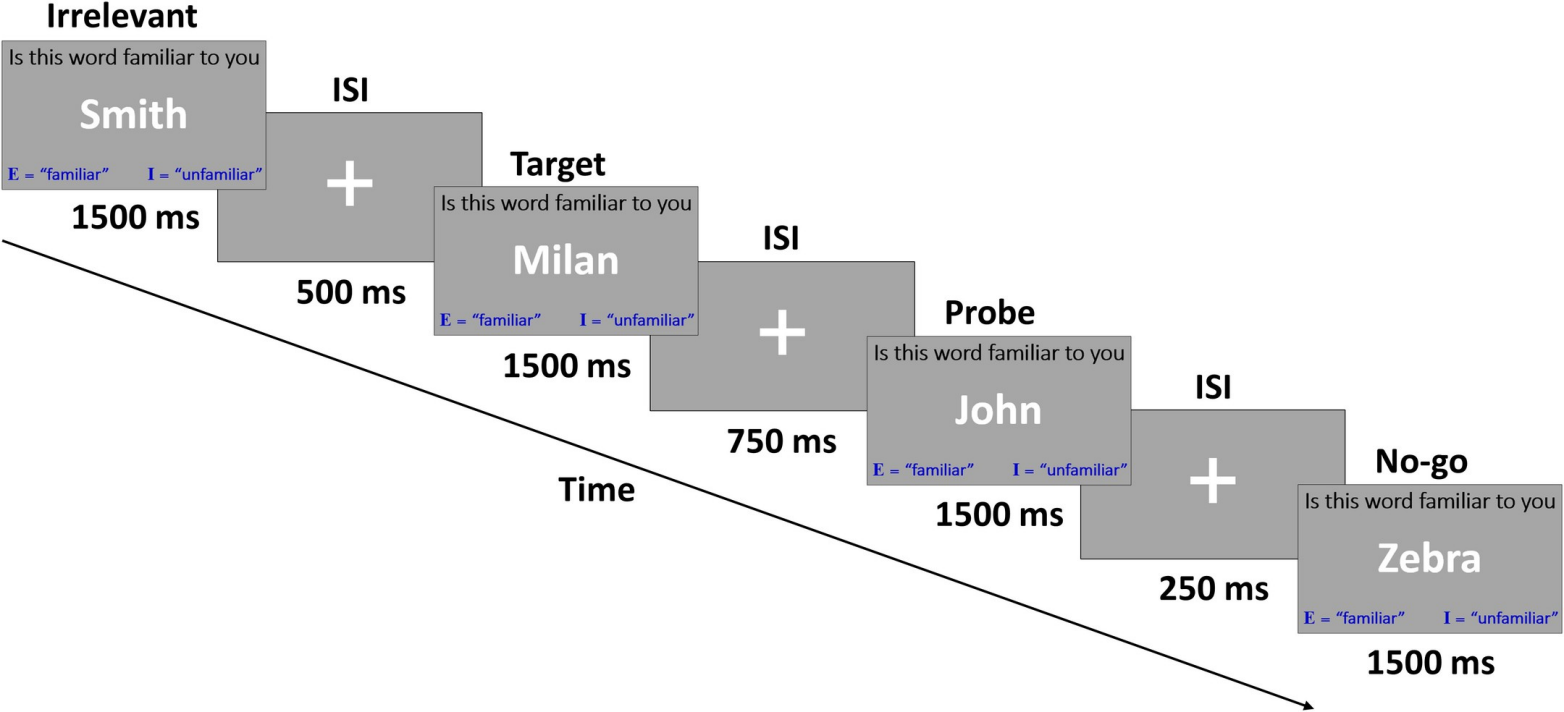
Experimental procedure of the novel Go/No-go RT-CIT. On “go” trials, which included irrelevant, probe and target items, participants had to respond by pressing a button. During “no-go” trials, participants were instructed not to respond.

The RT-CIT was operated according to the multiple-probes-protocol (MPP), which means that all 14 items were intermixed in each block of the CIT (there were 4 blocks in total). Per block, each item was presented 6 times, and thus, each block contained 84 items (14 x 6 = 84). The entire experiment contained 336 items (84 items x 4 blocks = 336). The order of items’ presentation was determined randomly, with the following restriction: two consecutive presentations of the same item were not allowed. All stimuli were displayed in a serial manner, in the middle of the screen, for 1500ms. Between each two items, a symbol of a plus was presented; this inter stimulus interval (ISI) was either 250ms, 500ms, or 750ms [similar to 28, 46, 86, 87]. On top of the items, participants also saw the question: "Is this word familiar to you"? Participants were requested to respond using one of two buttons: unfamiliar (i.e., “I”) for probes and irrelevant items, familiar (i.e., “E”) for targets [34, 88]. In addition, when seeing a no-go item, participants were requested not to respond. During ’go’ trials only, two feedback messages in the form of red words could briefly appear above the item for 200ms: (1) "WRONG" if participants pressed the wrong button, and (2) "TOO SLOW", if 800ms passed since the item appeared and no button was pressed [similar to 28, 38, 46, 86, 87, 89–91]. For a visual presentation, please see Fig 1.

Importantly, the actual RT-CIT was also preceded by three successive practice phases that familiarized participants with the test procedure. These practice phases were repeated until certain criteria were met (as detailed below). In the first practice phase, which included solely “go” trials, items remained on the screen until one of the two available buttons (“E” or “I”) was pressed. If participants pressed the wrong button, they received "WRONG" feedback. In the second practice phase, which included both “go” and “no-go” trials, items remained on the screen until a button was pressed or until 1500ms had elapsed. Similar to the first practice phase, participants received "WRONG" feedback in case of an incorrect response. In the last practice phase, participants also received "TOO SLOW" feedback if they failed to press any button within 800ms during “go” trials. Please note that participants were able to advance through each phase if they met the following three criteria: (1) a maximum of 50% errors (i.e., incorrect button presses), (2) a maximum of 20% of RTs falling under 150ms, and (3) a mean reaction time that did not exceed 800ms. If participants did not meet these criteria, they received feedback about their performance (i.e., "Sorry, you failed this practice phase. Please repeat the training") and had to perform the practice phase again (up to a maximum of two attempts).

#### Stage 3

After the CIT, participants were asked to complete the BIS-11 questionnaire. All items (a total of 30) were presented one by one, and participants were asked to rate their agreement for each item, on a scale from 1 (“rarely/never”) to 4 (“almost always"). Finally, after the BIS-11, participants were asked to complete four parts to summarize their experience in the experiment. First, they were asked to rate the significance level of the 2 probes, 2 targets, 8 irrelevants, and 2 no-go items on a scale from 1 ("not significant at all") to 9 ("extremely significant"). These ratings were obtained to examine (and ensure) that the selected probes were more significant than the irrelevant items. Second, participants were asked to rate how motivated they were to succeed in the test, on a scale from 1 ("not motivated at all") to 10 ("very motivated"). Third, participants were asked to rate how impulsive they think they were during the CIT, on a scale from 1 ("not impulsive at all") to 10 ("very impulsive"). Fourth, participants were presented with a list of countermeasures, and were asked to mark the options they used. If they didn’t use any countermeasures, they could mark the option "No countermeasures were used". At the end of the experiment, participants were thanked for their participation and granted their credit points.

#### Outliers and exclusions

Single items were excluded according to the following criteria: (1) Each button press under 150ms; (2) Each button press above 800ms; (3) Each error of pressing the wrong button.

Moreover, the data of an entire participant were excluded when: (1) The participant made at least 50% errors (in go trials of the CIT) to any of the 3 stimulus types (probe, irrelevant, target); (2) The participant did not complete the entire CIT (< 336 trials). Accordingly, all data of fourteen participants were excluded (see Participants).

## Results

All data were pre-processed using Matlab R2022b (The MathWorks, Natick, MA). Thereafter, data analyses were performed using JASP statistical program [92, version 0.17.2.]. The analysis plan was pre-registered on: https://osf.io/hz58u, and the data along with analysis scripts can be accessed at: https://osf.io/s5mrn/.

### Subjective ratings

Prior to testing the main hypothesis (i.e., correlation between the RT-CIT effect and secondary psychopathic traits), we analyzed the subjective ratings which were obtained after the CIT (these analyses were not pre-registered). First, we analyzed (1) participants self-reported motivation to conceal their identity during the CIT, and (2) participants self-reported impulsivity during the CIT (in both cases, scale ranged from 1–10). Both the motivation to conceal (*M* = 7.71, *SD* = 2.2) and experienced impulsivity (*M* = 6.22, *SD* = 2.16) were high.

Second, we analyzed the self-reported significance of probe and irrelevant items (scale ranged from 1–9). As expected, the significance of probes (*M* = 8.58, *SD* = 1.21) was higher than the significance of irrelevants (*M* = 1.94, *SD* = 1.1); *t*(85) = 36.01, *p* < .001, *d* = 3.88, BF_10_ = 9.440 × 10^+49^.

Third, we analyzed the reported countermeasures: 9% of participants reported that they tried to distract themselves; 14% reported that they tried to answer faster to the probe items (i.e., their own name); 1% reported that they tried to answer more slowly to probes; 2% reported that they tried to answer without looking at the screen; and 70% reported that they did not use any countermeasures.

### Main analyses

For the main analysis, we computed for each participant the RT-CIT effect, which is defined as the mean RT of probes minus the mean RT of irrelevants. As we relied on a modified RT-CIT with ‘no-go’ trials, we first compared the mean RT-CIT effect across participants (i.e., 55 ms; see also Table 1) to 0. A statistically significant difference was observed, *t*(85) = 15.7, *p* < .001, *d* = 1.69 (95% CI = [1.36, 2.02]), which was very strongly supported by the BF_10_ = 9.527×10^+23^.

**Table 1:** Preregistered measurement variables.

	RT-CIT effect	Mean probe RT	Mean irrelevant RT	Total LSRP score (26–104)	Primary LSRP score (16–64)	Secondary LSRP score (10–40)	BIS-11 score (30–120)	No-go errors
Mean	54.91	532.38	477.47	47.37	28.34	19.03	63.03	14.99
S.D.	32.43	37.31	33.28	7.03	5.33	3.26	10.23	7.49
Minimum	-23.98	439.52	375.95	31	18	11	46	2
Maximum	123.43	614.13	547.7	68	45	28	109	48

To test the main hypothesis, we correlated the individual RT-CIT effects with the secondary LSRP scores. Contrary to the research hypothesis, no significant correlation was observed: *r* = 0.04, *p* = 0.725, BF₀₁ = 6.98 (see Table 2).

**Table 2:** Correlations (r) and BFs between the RT-CIT effect, total LSRP score, primary LSRP score, secondary LSRP score, BIS-11 score and No-go error rate. All values below the diagonal are correlations (r), while all values above the diagonal are BFs.

Measure	RT-CIT effect	Total LSRP	Primary LSRP	Secondary LSRP	BIS-11	No-go errors
RT-CIT effect	—	7.134 (BF₀₁)	6.255 (BF₀₁)	6.984 (BF₀₁)	3.138 (BF₀₁)	3.077 (BF₀₁)
Total LSRP	-0.031	—	1.471×10^+28^ (BF₁₀)	5.115×10+^10^ (BF₁₀)	80.336 (BF₁₀)	3.937 (BF₀₁)
Primary LSRP	-0.064	0.897*	—	6.452 (BF₁₀)	1.764 (BF₀₁)	4.306 (BF₀₁)
Secondary LSRP	0.038	0.691*	0.300	—	43271.041 (BF₁₀)	5.724 (BF₀₁)
BIS-11	0.144	0.380*	0.185	0.516*	—	4.287 (BF₀₁)
No-go errors	0.146	0.124	0.115	0.079	0.115	—

Note: All significant correlations are marked with asterisks (* for p < .001; Bonferroni corrected alpha is 0.003)

**Fig 2:**
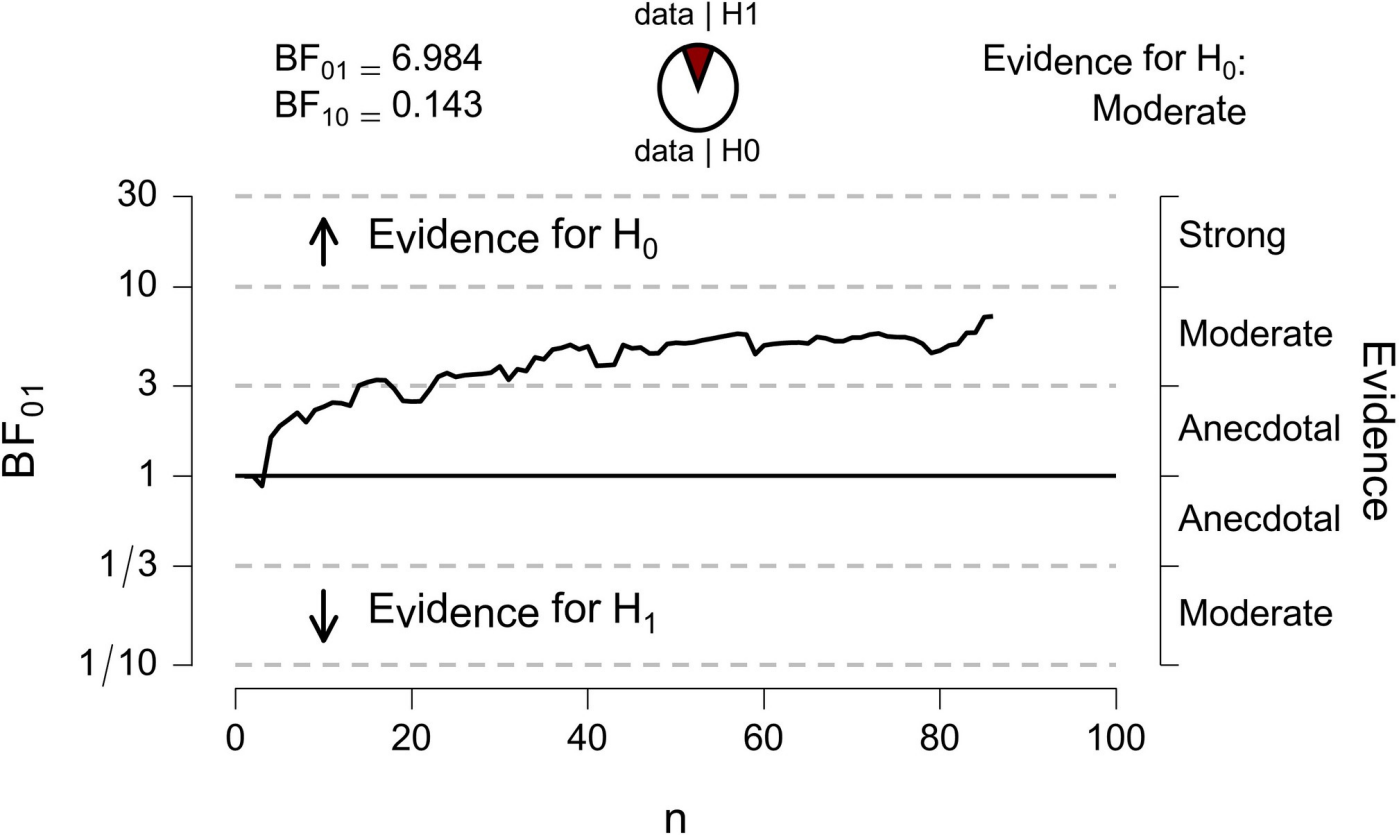
JASP output displaying a sequential analysis, showing the evidential flow of the null hypothesis (H_0_) versus the alternative hypothesis (H_1_), as data accumulates.

This result suggests that there is no linear association between the RT-CIT effect and secondary psychopathy (the null hypothesis is ~7 times more likely than the alternative hypothesis). Please note that similar results are obtained when including the data of the fourteen excluded participants: *r* = 0.03, *p* = 0.74, BF₀₁ = 7.5. Moreover, as can be seen in Fig 2, support for the null hypothesis increased as data accumulated.

To further examine the relationship between the RT-CIT effect, psychopathy, inhibition and impulsivity, we also correlated the RT-CIT effect with the total LSRP score, primary LSRP score, No-go error rate, and the BIS-11 score. Consistent with the main results reported above, which support the null hypothesis, no significant correlations were found with the RT-CIT effect (see Tables 1 and 2).

Notably, in a non-preregistered exploratory analysis, we performed a Bayesian Analysis of Covariance with Primary LSRP, Secondary LSRP, BIS-11, No-go errors, and Gender as predictors, and the RT-CIT effect as dependent variable. Using the BF_Inclusion_ metric, we compared all models including a particular predictor to those without the predictor [see 93]. The Inclusion BF for Secondary LSRP was 0.134 (note that similar values were observed for other predictors). This analysis further supports our main conclusion: there is no discernible linear relationship between secondary psychopathic traits and the RT-CIT effect (full results are available on the OSF at https://osf.io/s5mrn/).

### ROC analysis

As we relied on a novel CIT protocol, the area under the ROC curve (AUC) was calculated to measure the detection efficiency of classifying participants as unknowledgeable (naïve) or knowledgeable based on their individual probe-irrelevant score (i.e., the dCIT). The dCIT is computed by subtracting the mean RT of irrelevants from the mean RT of probes and dividing this difference by the standard deviation of irrelevant RTs [91]. As there were no naïve participants in the current experiment, their data were simulated. This simulation procedure is based on the assumption that naïve participants, in contrast to knowledgeable ones, cannot distinguish between probe and irrelevant items and therefore there is no reason to expect that probes would elicit systematic differential RTs. Thus, for naïve participants, the expected mean value of dCIT is 0. The standard deviation of dCIT was estimated using the following formula: $\frac{N-1}{N-3}*\frac{4}{N}\left(1\frac{\delta^{2}}{8}\right)$, where *N* is the total sample size and *δ* is the true effect size in the population, which is 0 in this case [e.g., 94]. Further, it was assumed that the data of individual naïve participants are distributed normally. Hence, a simulated dataset was created by taking *n* random samples from the normal distribution (with a mean of 0 and a SD computed as explained above). This simulation procedure, as well as the computation of the AUC, were repeated 1000 times using a bootstrapping procedure. These 1000 bootstrapped AUCs were then used to compute the mean AUC and its 95% confidence interval (CI). Accordingly, the mean AUC of our novel Go/No-go CIT was 0.92 (95% CI = [0.91, 0.94]), exceeding the average area (0.82) reported in the review paper by Meijer *et al*. (2016).

In sum, the novel CIT paradigm demonstrated impressive detection efficiency. However, contrary to our expectations, we observed no significant correlation between the RT-CIT effect and secondary psychopathic traits (BF_01_ = 6.98). This finding is further corroborated by the absence of significant correlations between the RT-CIT effect and both impulsivity (as measured by the BIS-11; BF₀₁ = 3.14) and response inhibition capacity (assessed by the no-go error rate; BF₀₁ = 3.08).

## Discussion

The present study examined the relation between the RT-CIT effect and secondary psychopathy in a student sample. The RT-CIT effect has been suggested to be largely driven by *response conflict* [28, 42, 46, 81]. Specifically, the need to classify familiar probes as “unfamiliar” induces a conflict. This conflict may be resolved by inhibiting the automatic “familiar” response, a process that consumes time and consequently slows down RT. Hence, it was hypothesized that individuals with higher secondary psychopathic traits, marked by impulsivity and impaired inhibition capacity, would produce larger RT-CIT effects compared to individuals with lower levels of secondary psychopathic traits.

Secondary psychopathic traits were measured using the LSRP questionnaire and correlated with the RT-CIT effect. Notably, both the mean score and reliability of the different LSRP scales were consistent with other reports in the literature [95–97]. Moreover, the mean RT-CIT effect was large and significantly different from 0 (Cohen’s *d* = 1.69; BF_10_ = 9.527×10^+23^). However, contrary to our hypothesis, no significant correlation between secondary psychopathy and the CIT effect was observed, as supported by the Bayesian analysis that revealed substantial evidence for the null hypothesis (BF_01_ = 6.98).

These findings are in line with those of Verschuere and in ´t Hout (2016), who examined the cognitive cost of lying among psychopaths using a Sheffield lie test (which measures deception, not concealed information). Similar to the present study, no significant correlation was found between psychopathy and the RT effect (RT_LIE_ minus RT_TRUTH_). Moreover, the current findings are in accordance with findings of CIT studies that used physiological measures and revealed no effect of psychopathy on the CIT [60, 62].

To delve deeper into our primary research question, we included two additional measures: *impulsivity* and *response inhibition* capacity. Impulsivity was assessed using the BIS-11 questionnaire, and although we found a significant correlation between impulsivity and secondary psychopathy, no significant correlation was observed between impulsivity and the RT-CIT effect [consistent with 81]. It is noteworthy that self-reports and behavioral measures (like the RT-CIT) typically yield weak correlations [97–101). Hence, to measure response inhibition capacity, we integrated a Go/No-go task within the CIT. However, consistent with our main findings, response inhibition capacity (as indicated by the no-go error rate) did not correlate with secondary psychopathy or the RT-CIT effect (please see Table 2).

Thus, the present study suggests that secondary psychopathy does not influence the RT-CIT effect. This conclusion should, however, be approached with caution for two primary reasons. Firstly, while our hypothesis was built on the premise that secondary psychopathy is marked by impulsivity and impaired response inhibition capacity, our measures of inhibition and secondary psychopathy did not correlate. This may be due to our non-forensic student sample. While studies utilizing non-forensic samples have generally shown no correlation between psychopathy and response inhibition capacity, studies involving forensic samples have demonstrated such a correlation [e.g., 102 vs. 103]. Secondly, our inhibition and CIT effect measures did not correlate. Although the integration of the Go/No-go task within the RT-CIT is unique to our study, few previous CIT studies have used “secondary response inhibition tasks”. For example, Ambach *et al*. (2008) included the Go/No-go task alongside the CIT (with different stimuli for each task, unlike the present study) and Suchotzki *et al*. (2019) introduced a Stroop task after the CIT. Both studies showed similar results to the present one–no significant correlation between response inhibition capacity and the RT-CIT effect. Ultimately, this raises the question of whether response conflict is the only mechanism underlying the RT-CIT.

Accordingly, as indicated previously, a recent study of klein Selle *et al*. (2023) has provided support for the idea that additional factors may contribute to the RT-CIT effect. These authors compared a *conflict* condition (where the response buttons emphasized familiarity) with a *no conflict* condition (where the response buttons emphasized categorical membership). Although conflict strengthened the RT-CIT effect, the effect was significant even in the no conflict condition. Therefore, it was suggested that conflict theory alone is not a sufficient account of the RT-CIT effect and that other mechanisms such as orientation may play a role. The orienting response entails reflexive behavioral and physiological responses to changes in the environment [104–106]. This response is primarily modulated by two key factors: the novelty of the stimulus and its perceived significance [70, 107, 108]. In the context of the CIT, probe items are both significant and novel (i.e., presented less frequently) for knowledgeable individuals. Hence, these items should elicit an enhanced orienting response. Such enhanced responses [103, 105] to significant probe items [see 63–66] may briefly interrupt ongoing behavior and consequently lengthen RTs. This notion is supported by a limited number of CIT studies. For instance, Lukács *et al*. (2019) categorized stimuli into three salience levels [forename, birthday, and favorite animal, from highest to lowest; 109] and found a larger RT-CIT effect for more significant items [91, 110]. Suchotzki *et al*. (2015) manipulated the proportion of probe versus irrelevant items and found a stronger RT-CIT effect for more novel probes [42].

Interestingly, when comparing our RT-CIT effect to that of a classical CIT study [46], which used a similar design, stimuli and was also performed online, a significant difference was observed. Specifically, the RT-CIT effect of our novel Go/No-go CIT, Cohen’s *d* = 1.69 (95% CI = [1.36, 2.02]), was significantly larger than that of the classical CIT study, Cohen’s *d* = 1.24 (95% CI [0.90; 1.57]). Although the BF (BF_10_ = 1.64) provides only weak evidence for this difference, a Bayesian sequential analysis showed increasing evidence for the alternative hypothesis as data accumulates (suggesting that more data should be obtained). Similarly, the Cohen’s *d* (1.69, 95% CI = [1.36, 2.02]) observed in the present study is higher than the mean Cohen’s *d* (1.30, 95% CI [1.06; 1.54]) reported in the meta-analysis of Suchotzki *et al*. (2017). Moreover, the current AUC value (0.92), which indicates detection efficiency of knowledgeable and unknowledgeable individuals, exceeds the mean AUC value (0.82) reported in the review paper by Meijer *et al*. (2016). Together this suggests that the additional “no-go” trials in our novel Go/No-go CIT may have increased CIT detection efficiency.

The observed increase in CIT detection efficiency may be the result of heightened cognitive load, a factor previously shown to enhance the RT-CIT effect [111–115]. For example, Visu-Petra *et al*. (2013) compared three CIT conditions: a classical RT-CIT, a RT-CIT with a concurrent memory task, and a RT-CIT with a concurrent set-shifting task. In line with the idea that additional cognitive load increases CIT detection efficiency, the RT-CIT effect was higher in the conditions that included an additional task (as evidenced by a larger increase in probe RTs than irrelevant RTs). Similarly, the no-go trials of our Go/No-go RT-CIT likely raised cognitive load, thereby reducing the capacity for inhibitory control and conflict resolution. Moreover, the additional no-go items may have also (1) made it harder to correctly respond to the different types of stimuli, thereby increasing conflict, and (2) diminished the relative frequency of probes, thereby amplifying the orienting response. As both conflict and orienting have been suggested to underlie the RT-CIT effect [see 46], it can explain how our modified format increased detection efficiency. Future investigations should aim to directly compare this novel format with a classical RT-CIT.

Additionally, while we strictly adhered to our preregistered protocol, future studies should aim to address several methodological limitations of the present study. First, as previously mentioned, the use of a non-forensic student sample may have influenced our findings. Therefore, investigating how more diverse samples could yield different results is essential. Moreover, conducting the experiment online may have influenced the RT-CT effect and, consequently, potentially affected the observed relationship between the RT-CIT effect and secondary psychopathy. Hence, replication studies conducted in a controlled laboratory setting are crucial [see 116]. Furthermore, while the use of highly salient autobiographical details ensured a strong CIT effect, it may not reflect real-world scenarios accurately. Thus, future studies should also examine the relationship between psychopathy and CIT using less salient crime-related stimuli, for instance. Lastly, it might be more appropriate to use the Single-Probe Protocol (SPP) of the CIT, where each block detects a single piece of information pertinent to the issue under investigation. This method is often the sole feasible interviewing approach in real-life contexts [117–120].

Furthermore, we would like to suggest that future examinations of psychopathy within the CIT incorporate both RT and neural measures. Notably, psychopaths exhibit distinct neural responses during tasks assessing conflict and orientation–i.e., the mechanisms assumed to underlie the RT-CT effect [121–128]. As such, methods such as fMRI, capable of monitoring conflict-related neural activity [see 42, 129–131], and EEG, capable of examining the P300 component of the event-related potential associated with attentional orientation [e.g., 132], hold particular promise. Integrating these neuroimaging methods would not only deepen our understanding of the RT-CIT effect but also further elucidate the neurobiological underpinnings of psychopathy, thereby advancing both fields of study.

In summary, previous studies have provided scientific evidence indicating that psychopathy does not affect the physiological response-based CIT [60, 62]. The present study provides preliminary evidence that psychopathic tendencies similarly do not affect the response time-based CIT. This is reassuring, as it suggests that although such tendencies do not improve CIT detection efficiency, they do not impede it. To expand and confirm these findings, future research is crucial. This should include conceptual replication studies using more diverse participant samples, CIT stimuli, and alternative protocols such as the SPP. Moreover, given the theoretical insight that orientation, alongside conflict, may drive the RT-CIT effect, it is imperative to thoroughly investigate the underlying mechanisms of this effect. Such exploration will not only advance theory but also deepen our understanding of practical aspects, such as susceptibility to countermeasures and potential influences from different clinical conditions. Ultimately, these investigations will bolster the validity and practical application of the RT-CIT across diverse settings and populations.

## Supporting information

S1 Graphical abstract (TIF)

## References

[1] Erat S, Gneezy U. White Lies. Management Science. 2012 Apr;58(4):723–33.

[2] Elaad E. The Distrusted Truth: Examination of Challenged Perceptions and Expectations. PSYCH. 2015;06(05):560–71.

[3] Saxe L. Lying: Thoughts of an applied social psychologist. American Psychologist. 1991 Apr;46(4):409–15.

[4] Verigin BL, Meijer EH, Bogaard G, Vrij A. Lie prevalence, lie characteristics and strategies of self-reported good liars. Sartori G, editor. PLoS ONE. 2019 Dec 3;14(12):e0225566. doi: 10.1371/journal.pone.0225566 31794563 PMC6890208

[5] Bond CF, DePaulo BM. Individual differences in judging deception: Accuracy and bias. Psychological Bulletin. 2008 Jul;134(4):477–92. doi: 10.1037/0033-2909.134.4.477 18605814

[6] Bond CF, DePaulo BM. Accuracy of Deception Judgments. Pers Soc Psychol Rev. 2006 Aug;10(3):214–34. doi: 10.1207/s15327957pspr1003_2 16859438

[7] Cook LG, Mitschow LC. Beyond the Polygraph: Deception Detection and the Autonomic Nervous System. Fed Pract. 2019 Jul;36(7):316–21. 31384120 PMC6654171

[8] Vrij A, Fisher RP. Which lie detection tools are ready for use in the criminal justice system? Journal of Applied Research in Memory and Cognition. 2016 Sep;5(3):302–7.

[9] Kleinmuntz B, Szucko JJ. Lie detection in ancient and modern times: A call for contemporary scientific study. American Psychologist. 1984;39(7):766–76. 6465664 10.1037//0003-066x.39.7.766

[10] Granhag PA, Vrij A, Verschuere B. Detecting deception: current challenges and cognitive approaches. Malden (Mass.): Wiley Blackwell; 2015. (Wiley series in the psychology of crime, policing and law).

[11] Ben-Shakhar G. Current Research and Potential Applications of the Concealed Information Test: An Overview. Front Psychology [Internet]. 2012 [cited 2023 May 29];3. Available from: http://journal.frontiersin.org/article/10.3389/fpsyg.2012.00342/abstract doi: 10.3389/fpsyg.2012.00342 23060826 PMC3462434

[12] Meijer EH, Verschuere B, Gamer M, Merckelbach H, Ben-Shakhar G. Deception detection with behavioral, autonomic, and neural measures: Conceptual and methodological considerations that warrant modesty: Deception research: Methodological considerations. Psychophysiol. 2016 May;53(5):593–604. 10.1111/psyp.12609 26787599

[13] Meijer EH, Selle NK, Elber L, Ben-Shakhar G. Memory detection with the Concealed Information Test: A meta analysis of skin conductance, respiration, heart rate, and P300 data: CIT meta-analysis of SCR, respiration, HR, and P300. Psychophysiol. 2014 Sep;51(9):879–904. 10.1111/psyp.12239 24916920

[14] Reid JE. A Revised Questioning Technique in Lie-Detection Tests. Journal of Criminal Law and Criminology (1931–1951). 1947 Mar;37(6):542. 20242527

[15] Ambach W, Gamer M. Physiological Measures in the Detection of Deception and Concealed Information. In: Detecting Concealed Information and Deception [Internet]. Elsevier; 2018 [cited 2022 Jul 4]. p. 3–33. Available from: https://linkinghub.elsevier.com/retrieve/pii/B978012812729200001X

[16] Ben-Shakhar G. A Critical Review of the Control Questions Test (CQT). In: Kleiner M, editor. Handbook of Polygraph Testing. Academic Press; 2002. p. 103–26.

[17] Committee to Review the Scientific Evidence on the Polygraph (National Research Council (U.S.)), National Research Council (U.S.), National Research Council (U.S.), National Research Council (U.S.), editors. The polygraph and lie detection. Washington, D.C: National Academies Press; 2003. 398 p.

[18] Bull R, Baron H, Gudjonsson G, Hampson S, Rippon G, Vrij A. A review of the current scientific status and fields of application of polygraphic deception detection. London, UK: British Psychological Society. 2004;

[19] Lewis JA, Cuppari M. The Polygraph: The Truth Lies within. The Journal of Psychiatry & Law. 2009 Mar;37(1):85–92.

[20] Iacono WG, Ben-Shakhar G. Current status of forensic lie detection with the comparison question technique: An update of the 2003 National Academy of Sciences report on polygraph testing. Law and Human Behavior. 2019 Feb;43(1):86–98. doi: 10.1037/lhb0000307 30284848

[21] Chan HC. Case 13—The Washington Green River Killer: The Case of Gary Leon Ridgway (1982–2001; U.S.A.). In: A Global Casebook of Sexual Homicide [Internet]. Singapore: Springer Singapore; 2019 [cited 2024 Jun 8]. p. 211–31. Available from: http://link.springer.com/10.1007/978-981-13-8859-0_14

[22] Rule A. Green River, running red: the real story of the Green River killer, America’s deadliest serial murderer. Gallery Books trade paperback edition. New York, NY: Gallery Books; 2004. 525 p.

[23] NITV Federal Services. NITV Federal Services. 2010 [cited 2024 Jun 8]. Killer passes polygraph, innocent man fails, killer goes on to kill again. Available from: https://www.cvsa1.com/press-releases/killer-passes-polygraph-innocent-man-fails-killer-goes-on-to-kill-again/

[24] Smith C, Guillén T. The Search for the Green River Killer: The True Story of America’s Most Prolific Serial Killer [Internet]. Open Road Media; 2017. Available from: https://books.google.co.il/books?id=DRAoDwAAQBAJ

[25] Lykken DT. The GSR in the detection of guilt. Journal of Applied Psychology. 1959;43(6):385–8.

[26] Verschuere B, Suchotzki K, Debey E. Detecting Deception Through Reaction Times. In: Granhag PA, Vrij A, Verschuere B, editors. Detecting Deception [Internet]. 1st ed. Wiley; 2014 [cited 2023 Sep 12]. p. 269–91. Available from: https://onlinelibrary.wiley.com/doi/10.1002/9781118510001.ch12

[27] Gamer M, Klimecki O, Bauermann T, Stoeter P, Vossel G. fMRI-activation patterns in the detection of concealed information rely on memory-related effects. Social Cognitive and Affective Neuroscience. 2012 Jun 1;7(5):506–15. doi: 10.1093/scan/nsp005 19258375 PMC3375883

[28] Suchotzki K, De Houwer J, Kleinberg B, Verschuere B. Using more different and more familiar targets improves the detection of concealed information. Acta Psychologica. 2018 Apr;185:65–71. doi: 10.1016/j.actpsy.2018.01.010 29407246

[29] Suchotzki K, Verschuere B, Van Bockstaele B, Ben-Shakhar G, Crombez G. Lying takes time: A meta-analysis on reaction time measures of deception. Psychological Bulletin. 2017;143(4):428–53. doi: 10.1037/bul0000087 28182460

[30] Varga M, Visu-Petra G, Miclea M, Buş I. The RT-based Concealed Information Test: An Overview of Current Research and Future Perspectives. Procedia—Social and Behavioral Sciences. 2014 Apr;127:681–5.

[31] Farwell LA, Donchin E. The Truth Will Out: Interrogative Polygraphy (“Lie Detection”) With Event-Related Brain Potentials. Psychophysiology. 1991 Sep;28(5):531–47. doi: 10.1111/j.1469-8986.1991.tb01990.x 1758929

[32] Sai L, Li H, Wang C, Rosenfeld JP, Lin X, Fu G. Feedback does not influence the recognition-related P300 in a novel concealed information test while feedback-evoked P300 shows promising diagnostic accuracy. International Journal of Psychophysiology. 2020 Nov;157:32–41. doi: 10.1016/j.ijpsycho.2020.08.003 32956772

[33] Rosenfeld JP. P300 in detecting concealed information. In: Verschuere B, Ben-Shakhar G, Meijer E, editors. Memory Detection [Internet]. 1st ed. Cambridge University Press; 2011 [cited 2022 Jun 29]. p. 63–89. Available from: https://www.cambridge.org/core/product/identifier/CBO9780511975196A017/type/book_part

[34] Suchotzki K, Verschuere B, Crombez G, De Houwer J. Reaction time measures in deception research: Comparing the effects of irrelevant and relevant stimulus–response compatibility. Acta Psychologica. 2013 Oct;144(2):224–31. doi: 10.1016/j.actpsy.2013.06.014 23920404

[35] Lukács G, Kleinberg B, Verschuere B. Familiarity-related fillers improve the validity of reaction time-based memory detection. Journal of Applied Research in Memory and Cognition. 2017 Sep;6(3):295–305.

[36] Verschuere B, De Houwer J. Detecting concealed information in less than a second: response latency-based measures. In: Verschuere B, Ben-Shakhar G, Meijer E, editors. Memory Detection [Internet]. 1st ed. Cambridge University Press; 2011 [cited 2022 Jun 29]. p. 46–62. Available from: https://www.cambridge.org/core/product/identifier/CBO9780511975196A016/type/book_part

[37] Lukács G, Ansorge U. Information leakage in the Response Time‐Based Concealed Information Test. Appl Cognit Psychol. 2019 Nov;33(6):1178–96.

[38] Lukács G, Ansorge U. The mechanism of filler items in the response time concealed information test. Psychological Research. 2021 Oct;85(7):2808–28. doi: 10.1007/s00426-020-01432-y 33449206 PMC8440312

[39] Mameli F, Sartori G, Scarpazza C, Zangrossi A, Pietrini P, Fumagalli M, et al. Honesty. In: Neuroimaging Personality, Social Cognition, and Character [Internet]. Elsevier; 2016 [cited 2023 May 29]. p. 305–22. Available from: https://linkinghub.elsevier.com/retrieve/pii/B9780128009352000166

[40] Seymour TL, Schumacher EH. Electromyographic evidence for response conflict in the exclude recognition task. Cognitive, Affective, & Behavioral Neuroscience. 2009 Mar 1;9(1):71–82. doi: 10.3758/CABN.9.1.71 19246328

[41] Walczyk JJ, Mahoney KT, Doverspike D, Griffith-Ross DA. Cognitive Lie Detection: Response Time and Consistency of Answers as Cues to Deception. J Bus Psychol. 2009 Mar;24(1):33–49.

[42] Suchotzki K, Verschuere B, Peth J, Crombez G, Gamer M. Manipulating item proportion and deception reveals crucial dissociation between behavioral, autonomic, and neural indices of concealed information: Concealed Information Test. Hum Brain Mapp. 2015 Feb;36(2):427–39. 25277495 10.1002/hbm.22637 PMC6869839

[43] Zheltyakova M, Kireev M, Korotkov A, Medvedev S. Neural mechanisms of deception in a social context: an fMRI replication study. Sci Rep. 2020 Dec;10(1):10713. doi: 10.1038/s41598-020-67721-z 32612101 PMC7329834

[44] Aron AR, Robbins TW, Poldrack RA. Inhibition and the right inferior frontal cortex: one decade on. Trends in Cognitive Sciences. 2014 Apr;18(4):177–85. doi: 10.1016/j.tics.2013.12.003 24440116

[45] Picton TW, Stuss DT, Alexander MP, Shallice T, Binns MA, Gillingham S. Effects of Focal Frontal Lesions on Response Inhibition. Cerebral Cortex. 2007 Apr;17(4):826–38. doi: 10.1093/cercor/bhk031 16699079

[46] klein Selle N, Or B, Van Der Cruyssen I, Verschuere B, Ben-Shakhar G. The role of response conflict in concealed information detection with reaction times. Sci Rep. 2023 Oct 19;13(1):17856. doi: 10.1038/s41598-023-43779-3 37857638 PMC10587134

[47] Gunschera LJ, Verschuere B, Murphy RA, Temple-McCune A, Dutton K, Fox E. No impaired integration in psychopathy: Evidence from an illusory conjunction paradigm. Personality Disorders: Theory, Research, and Treatment. 2023 Sep;14(5):479–89. doi: 10.1037/per0000619 37166836

[48] Verschuere B, Van Ghesel Grothe S, Waldorp L, Watts AL, Lilienfeld SO, Edens JF, et al. What features of psychopathy might be central? A network analysis of the Psychopathy Checklist-Revised (PCL-R) in three large samples. Journal of Abnormal Psychology. 2018 Jan;127(1):51–65. 29172600 10.1037/abn0000315

[49] De Brito SA, Forth AE, Baskin-Sommers AR, Brazil IA, Kimonis ER, Pardini D, et al. Psychopathy. Nat Rev Dis Primers. 2021 Jul 8;7(1):49. doi: 10.1038/s41572-021-00282-1 34238935

[50] van Dongen JDM. The Empathic Brain of Psychopaths: From Social Science to Neuroscience in Empathy. Front Psychol. 2020 Apr 16;11:695. doi: 10.3389/fpsyg.2020.00695 32477201 PMC7241099

[51] Karpman B. THE MYTH OF THE PSYCHOPATHIC PERSONALITY. AJP. 1948 Mar;104(9):523–34. doi: 10.1176/ajp.104.9.523 18911629

[52] Lykken DT. A study of anxiety in the sociopathic personality. The Journal of Abnormal and Social Psychology. 1957 Jul;55(1):6–10. doi: 10.1037/h0047232 13462652

[53] Sethi A, McCrory E, Puetz V, Hoffmann F, Knodt AR, Radtke SR, et al. Primary and Secondary Variants of Psychopathy in a Volunteer Sample Are Associated With Different Neurocognitive Mechanisms. Biological Psychiatry: Cognitive Neuroscience and Neuroimaging. 2018 Dec;3(12):1013–21. doi: 10.1016/j.bpsc.2018.04.002 29752216 PMC6185817

[54] Yildirim BO, Derksen JJL. Clarifying the heterogeneity in psychopathic samples: Towards a new continuum of primary and secondary psychopathy. Aggression and Violent Behavior. 2015 Sep;24:9–41.

[55] Cleckley HM. The Mask of Sanity. Postgraduate Medicine. 1976 Mar;9(3):193–7. 10.1080/00325481.1951.11694097 14807904

[56] Glenn AL. Early life predictors of callous-unemotional and psychopathic traits. Infant Ment Health J. 2019 Jan;40(1):39–53. doi: 10.1002/imhj.21757 30576592

[57] Hare RD. The Hare Psychopathy Checklist Revised (2nd ed.). Toronto: Multi-Health Systems; 2003.

[58] Dean AC, Altstein LL, Berman ME, Constans JI, Sugar CA, McCloskey MS. Secondary psychopathy, but not primary psychopathy, is associated with risky decision-making in noninstitutionalized young adults. Personality and Individual Differences. 2013 Jan;54(2):272–7. doi: 10.1016/j.paid.2012.09.009 23185100 PMC3505104

[59] Snowden RJ, Gray NS. Impulsivity and psychopathy: Associations between the Barrett Impulsivity Scale and the Psychopathy Checklist revised. Psychiatry Research. 2011 May;187(3):414–7. doi: 10.1016/j.psychres.2011.02.003 21377739

[60] Hong HG, Kim HS, Ji HK, Kim KP, Lee J, Jung SH, et al. Psychophysiological Responses of People with Psychopathic Tendencies to the Concealed Information Test. J Forensic Sci. 2018 May;63(3):766–70. doi: 10.1111/1556-4029.13600 28766714

[61] Verschuere B, in ´t Hout W. Psychopathic Traits and Their Relationship with the Cognitive Costs and Compulsive Nature of Lying in Offenders. Daunizeau J, editor. PLoS ONE. 2016 Jul 8;11(7):e0158595. doi: 10.1371/journal.pone.0158595 27391854 PMC4938600

[62] Verschuere B, Crombez G, De Clercq A, Koster EHW. Psychopathic traits and autonomic responding to concealed information in a prison sample. Psychophysiology. 2005 Mar;42(2):239–45. doi: 10.1111/j.1469-8986.2005.00279.x 15787861

[63] klein Selle N, Verschuere B, Kindt M, Meijer E, Ben-Shakhar G. Orienting versus inhibition in the Concealed Information Test: Different cognitive processes drive different physiological measures. Psychophysiology. 2016 Apr;53(4):579–90. doi: 10.1111/psyp.12583 26615984

[64] klein Selle N, Verschuere B, Kindt M, Meijer E, Ben-Shakhar G. Unraveling the roles of orienting and inhibition in the Concealed Information Test: Orienting and inhibition in the CIT. Psychophysiol. 2017 Apr;54(4):628–39. 10.1111/psyp.12825 28338233

[65] klein Selle N, Agari N, Ben-Shakhar G. Hide or Seek? Physiological Responses Reflect Both the Decision and the Attempt to Conceal Information. Psychol Sci. 2019 Oct;30(10):1424–33. doi: 10.1177/0956797619864598 31491366

[66] klein Selle N, Suchotzki K, Pertzov Y, Gamer M. Orienting versus inhibition: The theory behind the ocular‐based Concealed Information Test. Psychophysiology [Internet]. 2022 Oct 2 [cited 2022 Nov 14]; Available from: https://onlinelibrary.wiley.com/doi/10.1111/psyp.14186 36183237 10.1111/psyp.14186

[67] Levenson MR, Kiehl KA, Fitzpatrick CM. Assessing psychopathic attributes in a noninstitutionalized population. Journal of Personality and Social Psychology. 1995;68(1):151–8. doi: 10.1037//0022-3514.68.1.151 7861311

[68] Garofalo C, Noteborn MGC, Sellbom M, Bogaerts S. Factor Structure and Construct Validity of the Levenson Self-Report Psychopathy Scale (LSRP): A Replication and Extension in Dutch Nonclinical Participants. Journal of Personality Assessment. 2019 Sep 3;101(5):481–92. doi: 10.1080/00223891.2018.1519830 30362829

[69] Hare RD. Psychopathy as a risk factor for violence. Psychiatric Quarterly. 1999;70(3):181–97. doi: 10.1023/a:1022094925150 10457544

[70] Hare RD, Harpur TJ, Hakstian AR, Forth AE, Hart SD, Newman JP. The revised Psychopathy Checklist: Reliability and factor structure. Psychological Assessment: A Journal of Consulting and Clinical Psychology. 1990;2(3):338–41.

[71] Wang MC, Shou Y, Deng Q, Sellbom M, Salekin RT, Gao Y. Factor structure and construct validity of the Levenson Self-Report Psychopathy Scale (LSRP) in a sample of Chinese male inmates. Psychological Assessment. 2018 Jul;30(7):882–92. doi: 10.1037/pas0000537 29565613

[72] Bedwell SA, Hickman C. Effects of childhood trauma in psychopathy and response inhibition. Dev Psychopathol. 2022 Jan 25;1–6. doi: 10.1017/S0954579421001863 35074037

[73] Falkenbach D, Poythress N, Falki M, Manchak S. Reliability and Validity of Two Self-Report Measures of Psychopathy. Assessment. 2007 Dec;14(4):341–50. doi: 10.1177/1073191107305612 17986652

[74] Redondo N, Ronzón-Tirado R, Muñoz-Rivas MJ, Graña JL. Factor structure, psychometric properties, and proposal for a brief-form version of the Levenson Self-Report Psychopathy Scale: Validation in a court-referred partner-violent men sample. Personality and Individual Differences. 2023 Jul;208:112183.

[75] Trahair C, Baran L, Flakus M, Kowalski CM, Rogoza R. The structure of the Dark Triad traits: A network analysis. Personality and Individual Differences. 2020 Dec;167:110265.

[76] Meir-Levy R. The relationship between psychopathic features, attention functions, and achievements in lie detection tests. Bar-Ilan University; 2014.

[77] Wiemers EA, Redick TS. Task manipulation effects on the relationship between working memory and go/no-go task performance. Consciousness and Cognition. 2019 May;71:39–58. doi: 10.1016/j.concog.2019.03.006 30928898 PMC6488413

[78] Ambach W, Stark R, Peper M, Vaitl D. An interfering Go/No-go task does not affect accuracy in a Concealed Information Test. International Journal of Psychophysiology. 2008 Apr;68(1):6–16. doi: 10.1016/j.ijpsycho.2007.11.004 18180065

[79] Sebastian A, Pohl MF, Klöppel S, Feige B, Lange T, Stahl C, et al. Disentangling common and specific neural subprocesses of response inhibition. NeuroImage. 2013 Jan;64:601–15. doi: 10.1016/j.neuroimage.2012.09.020 22986077

[80] Patton JH, Stanford MS, Barratt ES. Factor structure of the barratt impulsiveness scale. J Clin Psychol. 1995 Nov;51(6):768–74. doi: 10.1002/1097-4679(199511)51:6<768::aid-jclp2270510607>3.0.co;2-1 8778124

[81] Suchotzki K, Kakavand A, Gamer M. Validity of the Reaction Time Concealed Information Test in a Prison Sample. Front Psychiatry. 2019 Jan 23;9:745. doi: 10.3389/fpsyt.2018.00745 30728785 PMC6351463

[82] Ergun S, Akca E, Yanartas O, Akca D, Ozercan A, Sayar K. The psychological determinants of emotional and external eating behavior in a university student sample from Turkey. Psihologija. 2022;(00):21–21.

[83] Lau JH, Jeyagurunathan A, Shafie S, Chang S, Samari E, Cetty L, et al. The factor structure of the Barratt Impulsiveness Scale (BIS-11) and correlates of impulsivity among outpatients with schizophrenia and other psychotic disorders in Singapore. BMC Psychiatry. 2022 Dec;22(1):226. doi: 10.1186/s12888-022-03870-x 35361174 PMC8968701

[84] Kramarski M. The effect of impulsiveness and self-monitoring on escalation of commitment. Bar-Ilan University; 2010.

[85] Peirce J, MacAskill M, Hirst B. Building experiments in psychopy. 2nd ed. Thousand Oaks: SAGE Publishing; 2022.

[86] Geven LM, Ben-Shakhar G, Kindt M, Verschuere B. It’s a match!? Appropriate item selection in the Concealed Information Test. Cogn Research. 2019 Dec;4(1):11. doi: 10.1186/s41235-019-0161-8 30945051 PMC6447635

[87] Geven LM, Ben‐Shakhar G, Kindt M, Verschuere B. Memory‐Based Deception Detection: Extending the Cognitive Signature of Lying From Instructed to Self‐Initiated Cheating. Topics in Cognitive Science. 2020 Apr;12(2):608–31. doi: 10.1111/tops.12353 29907999 PMC7379290

[88] Georgiadou K, Chronos A, Verschuere B, Sauerland M. Reaction time-based Concealed Information Test in eyewitness identification is moderated by picture similarity but not eyewitness cooperation. Psychological Research [Internet]. 2019 Jan 11 [cited 2022 Jul 22]; Available from: http://link.springer.com/10.1007/s00426-018-1139-8 10.1007/s00426-018-1139-8 PMC9470627 30635707

[89] Norman DG, Gunnell DA, Mrowiec AJ, Watson DG. Seen this scene? Scene recognition in the reaction-time Concealed Information Test. Mem Cogn. 2020 Nov;48(8):1388–402. doi: 10.3758/s13421-020-01063-z 32557195

[90] Kleinberg B, Verschuere B. The role of motivation to avoid detection in reaction time-based concealed information detection. Journal of Applied Research in Memory and Cognition. 2016 Mar;5(1):43–51.

[91] Kleinberg B, Verschuere B. Memory Detection 2.0: The First Web-Based Memory Detection Test. Ben Hamed S, editor. PLoS ONE. 2015 Apr 13;10(4):e0118715. doi: 10.1371/journal.pone.0118715 25874966 PMC4395266

[92] Wagenmakers EJ, Love J, Marsman M, Jamil T, Ly A, Verhagen J, et al. Bayesian inference for psychology. Part II: Example applications with JASP. Psychon Bull Rev. 2018 Feb;25(1):58–76. doi: 10.3758/s13423-017-1323-7 28685272 PMC5862926

[93] Van Den Bergh D, Van Doorn J, Marsman M, Draws T, Van Kesteren EJ, Derks K, et al. A Tutorial on Conducting and Interpreting a Bayesian ANOVA in JASP: L’Année psychologique. 2020 Feb 28;Vol. 120(1):73–96.

[94] Schmidt FL, Hunter JE. Methods of Meta-Analysis: Correcting Error and Bias in Research Findings [Internet]. 1 Oliver’s Yard, 55 City Road London EC1Y 1SP: SAGE Publications, Ltd; 2015 [cited 2023 Mar 12]. Available from: https://methods.sagepub.com/book/methods-of-meta-analysis-3e

[95] Culhane SE, Walker S, Hildebrand MM. Serial Homicide Perpetrators’ Self-Reported Psychopathy and Criminal Thinking. J Police Crim Psych. 2019 Mar;34(1):1–13.

[96] Walsh JA, Krienert JL, Thresher G, Potratz K. Examining the link between bullying participation, psychopathy and empathy in a large retrospective sample of university students. Criminal Justice Studies. 2018 Jul 3;31(3):249–66.

[97] Ye S, Yang Q, Lan T, Wang Y, Zhu B, Dong Y, et al. Psychopathic traits predict moral judgements in five moral domains: The mediating effect of unpleasantness. Leg Crim Psychol. 2021 Sep;26(2):176–95.

[98] Caswell AJ, Morgan MJ, Duka T. Inhibitory Control Contributes to “Motor”- but not “Cognitive”- Impulsivity. Experimental Psychology. 2013 Jun 1;60(5):324–34. doi: 10.1027/1618-3169/a000202 23628696

[99] Dang J, King KM, Inzlicht M. Why Are Self-Report and Behavioral Measures Weakly Correlated? Trends in Cognitive Sciences. 2020 Apr;24(4):267–9. doi: 10.1016/j.tics.2020.01.007 32160564 PMC7977810

[100] Ellingson JM, Potenza MN, Pearlson GD. Methodological factors as a potential source of discordance between self-report and behavioral measures of impulsivity and related constructs. Addictive Behaviors. 2018 Sep;84:126–30. doi: 10.1016/j.addbeh.2018.04.005 29660594 PMC5975131

[101] Malesza M, Ostaszewski P. Dark side of impulsivity—Associations between the Dark Triad, self-report and behavioral measures of impulsivity. Personality and Individual Differences. 2016 Jan;88:197–201.

[102] Weidacker K, Snowden RJ, Boy F, Johnston SJ. Response inhibition in the parametric Go/No-Go task in psychopathic offenders. Psychiatry Research. 2017 Apr;250:256–63. doi: 10.1016/j.psychres.2017.01.083 28171793

[103] Weidacker K, Whiteford S, Boy F, Johnston SJ. Response Inhibition in the Parametric Go/No-Go Task and Its Relation to Impulsivity and Subclinical Psychopathy. Quarterly Journal of Experimental Psychology. 2017 Mar;70(3):473–87. doi: 10.1080/17470218.2015.1135350 26821562

[104] Sokolov EN. Higher Nervous Functions: The Orienting Reflex. Annu Rev Physiol. 1963 Mar;25(1):545–80. doi: 10.1146/annurev.ph.25.030163.002553 13977960

[105] Sokolov EN. Orienting reflex as information regulator. In: Leontyev A, Luria A, Smirnov A, editors. Psychological Research in USSR. Moscow: Progress Publishers; 1966. p. 334–60.

[106] Verschuere B, Ben-Shakhar G. Theory of the Concealed Information Test. In: Verschuere B, Ben-Shakhar G, Meijer E, editors. Memory Detection [Internet]. 1st ed. Cambridge University Press; 2011 [cited 2023 Aug 5]. p. 128–48. Available from: https://www.cambridge.org/core/product/identifier/CBO9780511975196A020/type/book_part

[107] klein Selle N, Ben‐Shakhar G. A new theoretical perspective on concealed information detection. Psychophysiology [Internet]. 2022 Sep [cited 2023 Mar 18];60(3). Available from: https://onlinelibrary.wiley.com/doi/10.1111/psyp.14187 36166641 10.1111/psyp.14187 PMC10078248

[108] Gati I, Ben-Shakhar G, Avni-Liberty S. Stimulus novelty and significance in electrodermal orienting responses: The effects of adding versus deleting stimulus components. Psychophysiology. 1996 Nov;33(6):637–43. doi: 10.1111/j.1469-8986.1996.tb02358.x 8961784

[109] Lukács G, Grządziel A, Kempkes M, Ansorge U. Item Roles Explored in a Modified P300-Based CTP Concealed Information Test. Appl Psychophysiol Biofeedback. 2019 Sep;44(3):195–209. doi: 10.1007/s10484-019-09430-6 30969387 PMC6685925

[110] Verschuere B, Kleinberg B, Theocharidou K. RT-based memory detection: Item saliency effects in the single-probe and the multiple-probe protocol. Journal of Applied Research in Memory and Cognition. 2015 Mar;4(1):59–65.

[111] Ambach W, Stark R, Vaitl D. An interfering n-back task facilitates the detection of concealed information with EDA but impedes it with cardiopulmonary physiology. International Journal of Psychophysiology. 2011 Jun;80(3):217–26. doi: 10.1016/j.ijpsycho.2011.03.010 21440579

[112] Debey E, Verschuere B, Crombez G. Lying and executive control: An experimental investigation using ego depletion and goal neglect. Acta Psychologica. 2012 Jun;140(2):133–41. doi: 10.1016/j.actpsy.2012.03.004 22627157

[113] Hu X, Evans A, Wu H, Lee K, Fu G. An interfering dot-probe task facilitates the detection of mock crime memory in a reaction time (RT)-based concealed information test. Acta Psychologica. 2013 Feb;142(2):278–85. doi: 10.1016/j.actpsy.2012.12.006 23376139

[114] Liang J, Chen Y, Yan W, He Y. Enhanced detection efficiency in reaction time‐based concealed information test through response preparation interference. Applied Cognitive Psychology. 2024 Jan;38(1):e4180.

[115] Visu-Petra G, Varga M, Miclea M, Visu-Petra L. When Interference Helps: Increasing Executive Load to Facilitate Deception Detection in the Concealed Information Test. Front Psychol [Internet]. 2013 [cited 2023 Mar 20];4. Available from: http://journal.frontiersin.org/article/10.3389/fpsyg.2013.00146/abstract doi: 10.3389/fpsyg.2013.00146 23543918 PMC3610081

[116] Crump MJC, McDonnell JV, Gureckis TM. Evaluating Amazon’s Mechanical Turk as a Tool for Experimental Behavioral Research. Gilbert S, editor. PLoS ONE. 2013 Mar 13;8(3):e57410. doi: 10.1371/journal.pone.0057410 23516406 PMC3596391

[117] Koller D, Hofer F, Grolig T, Ghelfi S, Verschuere B. What are you hiding? Initial validation of the reaction time‐based searching concealed information test. Applied Cognitive Psychology. 2020 Nov;34(6):1406–18.

[118] Ogawa T, Matsuda I, Tsuneoka M, Verschuere B. The Concealed Information Test in the Laboratory Versus Japanese Field Practice: Bridging the Scientist–Practitioner Gap. Archives of Forensic Psychology. 2015;1(2):16–27.

[119] Podlesny JA. A paucity of operable case facts restricts applicability of the guilty knowledge technique in FBI criminal polygraph examinations. Forensic Science Communications [Internet]. 2003 [cited 2024 Jun 23];5(3). Available from: https://www.ojp.gov/ncjrs/virtual-library/abstracts/paucity-operable-case-facts-restricts-applicability-guilty

[120] Rosenfeld JP, Shue E, Singer E. Single versus multiple probe blocks of P300-based concealed information tests for self-referring versus incidentally obtained information. Biological Psychology. 2007 Mar;74(3):396–404. doi: 10.1016/j.biopsycho.2006.10.002 17126984

[121] Clark AP, Bontemps AP, Batky BD, Watts EK, Salekin RT. Psychopathy and neurodynamic brain functioning: A review of EEG research. Neuroscience & Biobehavioral Reviews. 2019 Aug;103:352–73. doi: 10.1016/j.neubiorev.2019.05.025 31158388

[122] Brazil IA, Verkes RJ, Brouns BHJ, Buitelaar JK, Bulten BH, De Bruijn ERA. Differentiating Psychopathy from General Antisociality Using the P3 as a Psychophysiological Correlate of Attentional Allocation. Fontenelle L, editor. PLoS ONE. 2012 Nov 16;7(11):e50339. doi: 10.1371/journal.pone.0050339 23166843 PMC3500377

[123] Verona E, Sprague J, Sadeh N. Inhibitory control and negative emotional processing in psychopathy and antisocial personality disorder. Journal of Abnormal Psychology. 2012 May;121(2):498–510. doi: 10.1037/a0025308 22288907

[124] Gao Y, Zhang W, Eisenbarth H, Fung ALC, Lu M, Raine A, et al. P3 amplitude and psychopathic traits in youths: Distinct contributions of the grandiose-manipulative and daring-impulsivity traits. Personality and Individual Differences. 2018 Jan;120:87–94.

[125] Anton ME, Baskin-Sommers AR, Vitale JE, Curtin JJ, Newman JP. Differential effects of psychopathy and antisocial personality disorder symptoms on cognitive and fear processing in female offenders. Cogn Affect Behav Neurosci. 2012 Dec;12(4):761–76. doi: 10.3758/s13415-012-0114-x 22886692 PMC3508139

[126] Anderson NE. Functional neuroimaging and psychopathy. In: Kiehl KA, Sinnott-Armstrong WP, editors. Handbook on Psychopathy and Law. Oxford University Press; 2013. p. 131–49.

[127] Krakowski MI, Hoptman MJ, Czobor P. Neural Correlates of Psychopathic Traits in Schizophrenia: fMRI Study of Response Inhibition in Persistently Violent Patients. Schizophrenia Bulletin Open. 2023 Jan 1;4(1):sgad009. doi: 10.1093/schizbullopen/sgad009 39145336 PMC11207843

[128] Laurens KR, Kiehl KA, Smith AM, Forster BB, Liddle PF. Abnormal response inhibition in criminal psychopaths: Evidence from event-related fMRI. NeuroImage. 2001 Jun;13(6):1068.

[129] Hsu C, Begliomini C, Dall’Acqua T, Ganis G. The effect of mental countermeasures on neuroimaging‐based concealed information tests. Human Brain Mapping. 2019 Jul;40(10):2899–916. doi: 10.1002/hbm.24567 30864277 PMC6865496

[130] Gamer M, Bauermann T, Stoeter P, Vossel G. Covariations among fMRI, skin conductance, and behavioral data during processing of concealed information. Human Brain Mapping. 2007 Dec;28(12):1287–301. doi: 10.1002/hbm.20343 17290371 PMC6871443

[131] Gamer M. Mind Reading Using Neuroimaging: Is This the Future of Deception Detection? European Psychologist. 2014 Jan 1;19(3):172–83.

[132] klein Selle N, Gueta C, Harpaz Y, Deouell LY, Ben-Shakhar G. Brain-based concealed memory detection is driven mainly by orientation to salient items. Cortex. 2021 Mar;136:41–55. doi: 10.1016/j.cortex.2020.12.010 33460912

