# Supplementary figures and images for: Fast & furious: Rejecting the hypothesis that secondary psychopathy improves reaction time-based concealed information detection
Source: PLoS One. 2024 Oct 15;19(10):e0311948. doi: 10.1371/journal.pone.0311948 (PMC11478853; doi:10.1371/journal.pone.0311948)

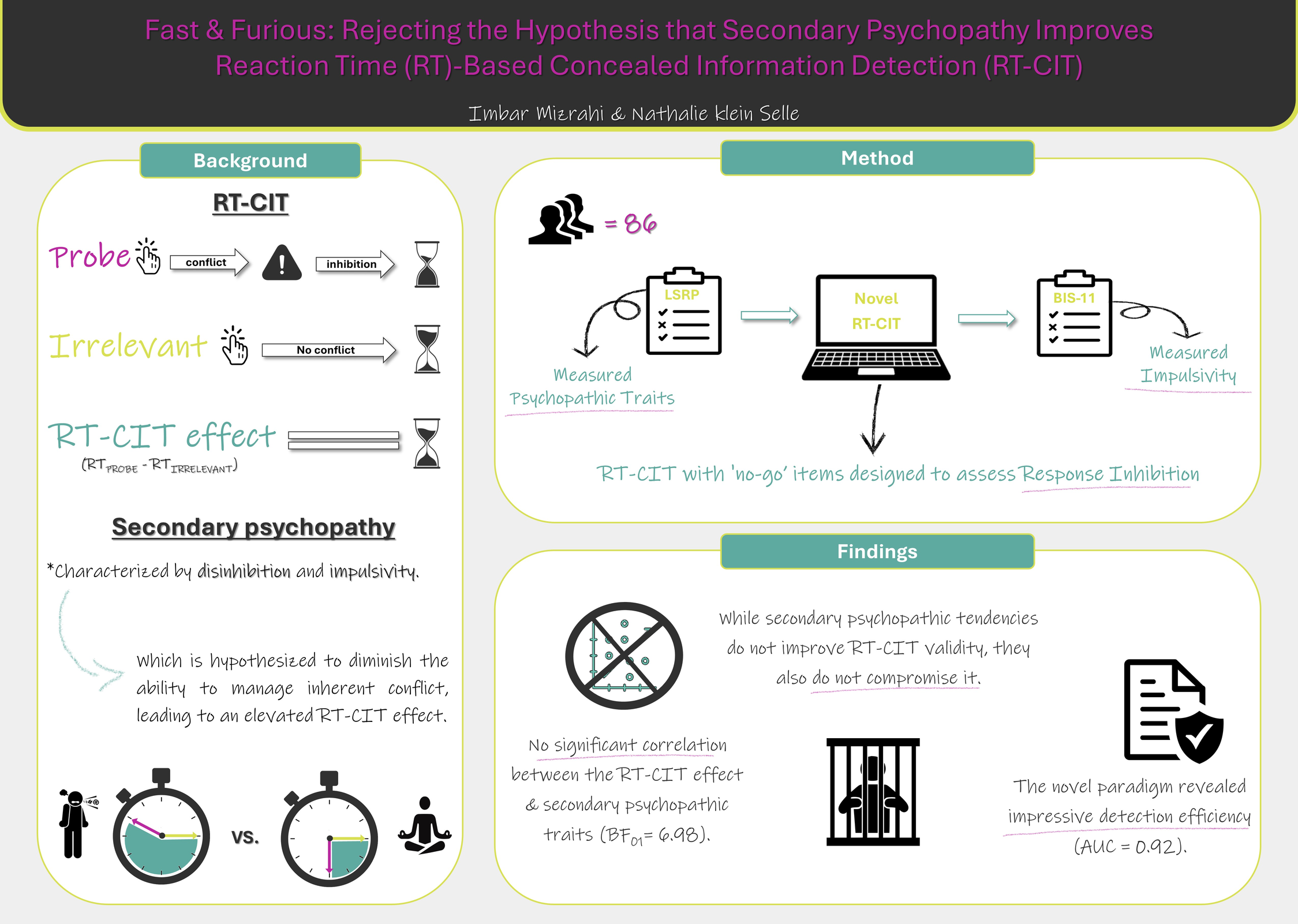

Supplement: S1 Graphical abstract — (TIF) [file pone.0311948.s001.tif]
